# Author´s reply to comment on: Prenatal exposure to paracetamol and risk of autism spectrum disorder: systematic review and meta-analysis of observational studies

**DOI:** 10.61622/rbgo/2026rbgo108AR1

**Published:** 2026-09-23

**Authors:** Juliana Almeida Oliveira, Maria Julia Lemos, Laura Fonseca Queiroz, João Vitor Sciorilli, Tam Tran, Isabella de Oliveira Ortiz, Bruna Benigna Sales Armstrong, Flávia Ribeiro de Oliveira

**Affiliations:** 1 Universidade Federal de Minas Gerais Department of Surgery Belo Horizonte MG Brazil Department of Surgery, Universidade Federal de Minas Gerais, Belo Horizonte, MG, Brazil.; 2 Centro Universitário Redentor Itaperuna RJ Brazil Centro Universitário Redentor, Itaperuna, RJ, Brazil.; 3 Santa Casa de Misericórdia de Belo Horizonte Department of Obstetrics and Gynecology Belo Horizonte MG Brazil Department of Obstetrics and Gynecology, Santa Casa de Misericórdia de Belo Horizonte, Belo Horizonte, MG, Brazil.; 4 Centro Universitário Faculdade de Medicina do ABC Santo André SP Brazil Centro Universitário Faculdade de Medicina do ABC, Santo André, SP, Brazil.; 5 Washington University School of Medicine Saint Louis MO United States of America Washington University School of Medicine, Saint Louis, MO, United States of America.; 6 Hospital Santa Marcelina São Paulo SP Brazil Hospital Santa Marcelina, São Paulo, SP, Brazil.; 7 Irmandade Santa Casa de Misericórdia São Paulo SP Brazil Irmandade Santa Casa de Misericórdia, São Paulo, SP, Brazil.; 8 Universidade Federal de Minas Gerais Department of Obstetrics and Gynecology Belo Horizonte MG Brazil Department of Obstetrics and Gynecology, Universidade Federal de Minas Gerais, Belo Horizonte, MG, Brazil.

Dear Editor,

We thank the correspondents for their careful appraisal of our systematic review and meta-analysis. Their comments led to a study-level audit of data provenance, effect-estimate selection, and the statistical analyses. We appreciate the opportunity to clarify our methods and refine the interpretation of our findings.

A central concern raised in the correspondence relates to the study by Ahlqvist et al., which contributed 95.7% of the statistical weight in the primary analysis.^(1)^ The large contribution of this study reflects its substantially larger sample size and corresponding statistical precision, as expected in inverse-variance meta-analysis. Nevertheless, because a highly weighted study may raise legitimate concerns regarding the robustness of a pooled estimate, we performed a leave-one-out sensitivity analysis in which each study was sequentially excluded and the meta-analysis repeated. Exclusion of Ahlqvist et al. yielded a pooled OR of 1.28 (95% CI 1.02–1.60; p = 0.031). The direction of the association was preserved, although precision decreased and heterogeneity increased (from 45.9% to 58.3%) after exclusion of the largest study, with statistical significance maintenance (p = 0.0311). Similarly, sequential exclusion of the remaining individual studies did not alter the direction or statistical significance of the pooled estimate. Thus, although Ahlqvist et al. contributed the greatest statistical weight, the observed association was not dependent exclusively on this study.^(2)^

The correspondence also correctly notes that an OR of 1.18 is not explicitly reported in the Ahlqvist et al. publication.^(1)^ This estimate was not extracted as a reported OR; rather, it was calculated from the published event data to obtain an effect estimate on the odds-ratio scale for the quantitative synthesis. Ahlqvist et al. reported 5,857 ASD diagnoses among children exposed to acetaminophen during pregnancy, and 3,956 in the sibling cohort; compared with 61,459 diagnoses among unexposed children, and 41,294 in the sibling cohort.^(1)^ These published data were analyzed in R using the meta package to derive the OR included in our analysis.

Deriving a common effect measure from sufficiently reported group-level data is an established approach in evidence synthesis. The Cochrane Handbook for Systematic Reviews of Interventions describes methods for calculating effect estimates from published data when the necessary information is available, allowing studies to be expressed using a common measure for quantitative synthesis.^(3-5)^ We recognize, however, that our original report did not describe the derivation of this estimate with sufficient specificity.

Our audit identified an important methodological distinction that was insufficiently explicit in the original report. The reconstructed OR for Ahlqvist et al. is an unadjusted measure based on cumulative event counts, whereas the original investigators analyzed ASD as a time-to-event outcome and reported an adjusted population-based HR of 1.05 (95% CI 1.02–1.08) and a sibling-comparison HR of 0.98 (95% CI 0.93–1.04). These measures are not interchangeable. The reconstructed OR does not preserve the covariate adjustment, follow-up time, or censoring structure of the original survival analysis. Because this reconstructed estimate contributed 95.7% of the statistical weight, its limitations materially affect the degree of certainty that can be attached to the original pooled OR.

These findings reduce the degree of certainty that should be attached to the original pooled OR. Conventional observational analyses remain compatible with a small association, but the magnitude and independence of that association are uncertain, and the available evidence does not establish a causal effect. This interpretation is reinforced by the full-sibling analysis of Ahlqvist et al.,^(1)^ in which the association was attenuated to the null. Familial factors, confounding by indication, and other sources of residual confounding should therefore receive greater emphasis when interpreting the evidence.

The audit also confirmed that HRs, IRRs, and ORs were not directly pooled as interchangeable measures in the original analysis. Nevertheless, reconstructing ORs from aggregate event counts may result in loss of covariate adjustment and time-to-event information. This limitation should have been explicitly reported. Numerical information recovered from graphical sources was presented in the Supplementary Material for transparency but was not entered as an effect estimate in the quantitative synthesis. Furthermore, numerical information extracted from figures using PlotDigitizer were included in the Supplementary Material for transparency but were not used as an effect estimate in the quantitative meta-analysis.^(6)^ We agree, however, that supplementary presentation should make clear which data represent original reported measures, which were calculated from published data, and which were recovered from graphical sources.

To further assess whether the selection of study-level effect estimates materially influenced the pooled result, we performed an additional sensitivity analysis using the unadjusted ASD estimate explicitly reported by Ji et al. (OR 1.94).^(7)^ The pooled estimate remained essentially unchanged (OR 1.18, 95% CI 1.15–1.21; p < 0.001), with low between-study heterogeneity (I^2^ = 13%; Cochran's Q p = 0.3277). This analysis provides additional evidence that the overall finding was not materially dependent on the specific effect estimate initially selected for Ji et al.^(7)^

Regarding the Generation R cohort, Snijder et al.^(8)^ was not used as the source of an ASD effect estimate. That publication reported earlier non-ASD outcomes from the cohort, including cryptorchidism and hypospadias. ASD-related information from this population was obtained from the subsequent publication by Alemany et al.^(9)^ We acknowledge that the distinction between the cohort-profile or earlier cohort publications and the publications providing the ASD estimates from the original authors could have been made clearer in the Supplementary Material.

As part of the audit, we constructed a cohort-by-publication matrix to verify the provenance of each estimate and assess potential population overlap. This review confirmed that previous meta-analyses were not treated as independent studies and that the same underlying cohort did not contribute more than once to the same quantitative synthesis.

We additionally confirm a transcription error in the Results section. The correct confidence interval for the original pooled OR is 1.15–1.21, as displayed in the forest plot, rather than 1.15–1.32. We will request a formal correction addressing this reporting error, the derivation and limitations of the reconstructed effect estimate, and the clarification of data provenance in the Supplementary Material. Where appropriate, the wording of the Results, Discussion, and Conclusions will also be revised to reflect the greater uncertainty identified by the methodological reassessment.

Finally, we highlight again that our findings should not be interpreted as demonstrating that prenatal acetaminophen exposure causes ASD or as supporting avoidance of acetaminophen when clinically indicated during pregnancy. Available evidence is observational and remains vulnerable to confounding by indication, shared familial factors, and other residual confounding. Clinical decisions should remain individualized and based on the balance between the benefits and risks of treatment. Accordingly, our clinical interpretation is one of judicious and individualized use rather than generalized avoidance. Contemporary regulatory and professional guidance similarly emphasizes that the available evidence does not establish causality and that acetaminophen remains an appropriate therapeutic option during pregnancy when clinically indicated. The U.S. Food and Drug Administration has acknowledged evidence of a possible association while emphasizing the uncertainty regarding causality.^(10)^ The American College of Obstetricians and Gynecologists supports judicious use, including the lowest effective dose for the shortest necessary duration when treatment is clinically indicated.^(11)^ The World Health Organization has likewise stated that there is no conclusive scientific evidence establishing a causal link between acetaminophen use during pregnancy and autism.^(12)^ Our recommendation should therefore be understood as supporting individualized benefit-risk assessment and avoidance of unnecessary medication exposure, not avoidance of clinically indicated treatment.

We thank the correspondents for prompting this methodological reassessment. Their comments allowed us to improve the transparency of our analysis and, importantly, to distinguish more clearly between the mathematical reproducibility of a statistical association and evidence sufficient to support causal inference.

## Data Availability

The research data are described in the letter presented.
